# Genome-Wide Identification and Characterization of Cucumber BPC Transcription Factors and Their Responses to Abiotic Stresses and Exogenous Phytohormones

**DOI:** 10.3390/ijms20205048

**Published:** 2019-10-11

**Authors:** Shuzhen Li, Li Miao, Bin Huang, Lihong Gao, Chaoxing He, Yan Yan, Jun Wang, Xianchang Yu, Yansu Li

**Affiliations:** 1 Institute of Vegetables and Flowers, Chinese Academy of Agricultural Sciences, Beijing 100081, China; limengfbj@126.com (S.L.); happymml@163.com (L.M.); 82101175049@caas.cn (B.H.); hechaoxing@caas.cn (C.H.); yanyan@caas.cn (Y.Y.); wangjun01@caas.cn (J.W.); 2 Beijing Key Laboratory of Growth and Developmental Regulation for Protected Vegetable Crops, College of Horticulture, China Agricultural University, Beijing 100193, China; gaolh@cau.edu.cn

**Keywords:** BASIC PENTACYSTEINE (BPC), cucumber, expression analysis, abiotic stress, plant hormones

## Abstract

BASIC PENTACYSTEINE (BPC) is a small transcription factor family that functions in diverse growth and development processes in plants. However, the roles of BPCs in plants, especially cucumber (*Cucumis sativus* L.), in response to abiotic stress and exogenous phytohormones are still unclear. Here, we identified four BPC genes in the cucumber genome, and classified them into two groups according to phylogenetic analysis. We also investigated the gene structures and detected five conserved motifs in these *CsBPCs*. Tissue expression pattern analysis revealed that the four *CsBPCs* were expressed ubiquitously in both vegetative and reproductive organs. Additionally, the transcriptional levels of the four *CsBPCs* were induced by various abiotic stress and hormone treatments. Overexpression of *CsBPC2* in tobacco (*Nicotiana tabacum*) inhibited seed germination under saline, polyethylene glycol, and abscisic acid (ABA) conditions. The results suggest that the CsBPC genes may play crucial roles in cucumber growth and development, as well as responses to abiotic stresses and plant hormones. *CsBPC2* overexpression in tobacco negatively affected seed germination under hyperosmotic conditions. Additionally, *CsBPC2* functioned in ABA-inhibited seed germination and hypersensitivity to ABA-mediated responses. Our results provide fundamental information for further research on the biological functions of BPCs in development and abiotic stress responses in cucumber and other plant species.

## 1. Introduction

Cucumber (*Cucumis sativus* L.) is a major vegetable crop worldwide and has served as a model system for studies on sex determination [1] and plant vascular biology [2]. However, its growth and development are frequently affected by various stresses, such as low temperature [3], high salinity [4,5], water deficit [6], and pathogen attack [7], which severely reduce production and quality. Therefore, functional studies of cucumber stress responses and identification of stress-related genes are required to elucidate the molecular mechanisms of cucumber stress tolerance and protect them from detrimental surroundings.

BASIC PENTACYSTEINE (BPC)/BARLEY B RECOMBINANT (BBR), a plant-specific transcription factor family, is characterized by the ability to bind gene promoter sequences at the GAGA motif; the soybean GAGA-binding protein (GBP) binds to a (GA)_9_ repeat sequence of the *Glutamate 1-Semialdehyde Aminotransferase* (*Gsa1*) promoter [8], the barley *BARLEY B RECOMBINANT* (*BBR*) factor binds specifically to the (GA)_8_ repeat in vitro [9], and *Arabidopsis* BPC proteins recognize (GA)_6_ and (GA)_9_ sequences [10,11]. According this characteristic, BPC transcription factors are also named GAGA-binding transcriptional activators. To date, based on gene sequence similarity and protein domain structures, seven *Arabidopsis* BPC genes have been identified and classified into three classes: class I (BPC1–3), class II (BPC4–6), and class III (BPC7). All of the BPC proteins contain a highly conserved DNA-binding domain with five conserved cysteine residues at their C terminus [10]. Among these genes, BPC5 is thought to be a pseudogene that is unable to produce an active protein [10,12].

As newly identified transcription factors, BPCs have been proposed to function in diverse plant growth and development responses. For example, transcripts of the barley BBR gene were detected in all tissues, including roots, stems, leaves, inflorescences, and embryos, among which the highest level was observed in embryos and the lowest in leaves. Overexpression of the gene in tobacco resulted in pronounced leaf and flower shapes or structural modifications [9]. In *Arabidopsis*, all seven BPC genes except for *BPC5* were expressed ubiquitously in both vegetative and reproductive organs. Multiple *BPC* allele mutants displayed pleiotropic developmental defects, including dwarfism, small rosettes, early flowering, aberrant ovules, unopened floral buds, and even high sterility [12,13,14,15]. The reason for these morphological changes may be that BPCs bind to and regulate the activities and expression levels of target genes associated with development. BPC genes have been reported to be regulators of *INNER NO OUTER* (*INO*), a gene involved in ovule development [10]. BPC genes were also found to regulate the expression of the homeotic MADS box gene *SEEDSTICK* (*STK*), to control the ovule identity [11,13,16,17]. The *LEAFY COTYLEDON 2* (*LEC2*) gene is only expressed in embryos and acts as a master regulator of seed development, and is regulated by BPCs in *Arabidopsis* [18,19,20]. Additionally, BPCs downregulated the expression of the genes *SHOOTMERISTEMLESS* (*STM*) and *BREVIPEDICELLUS/KNAT1* (*BP*), which resulted in floral organ malformation [14]. Additionally, class I BPCs also act as direct regulators of several HOMEOBOX genes, such as *KNOX*, *WUS,* and *BELL* family members [14]. In our previous research, we found that cucumber BPCs are involved in seed germination as regulators of *ABSCISIC ACID INSENSITIVE3* (*ABI3*) [21]. In rice, the GAGA-binding transcription factors *OsGBP1* and *OsGBP3* displayed functional divergence in the regulation of grain size and plant growth [22]. *OsGBP1* also delayed flowering time by directly binding to the promoter of *OsLFL1*, a LEC2/FUS3-like gene, which constitutively inhibits the expression of a flowering activator, *Early heading date 1* (*Ehd1*) [22,23,24]. Moreover, increasing evidence has also demonstrated a role for BPCs in the regulation of plant responses to hormones, such as ethylene [12] and cytokinins [14,25]. While BPCs are known to participate in numerous developmental processes, the involvement of BPCs in stress responses is not clear.

The completion of genome sequencing for more species has enabled many genes to be identified and characterized. However, comprehensive analyses of BPC genes in cucumber and other plant species are still limited. Thus, in the present study, we identified four *BPC* family members in the cucumber genome, named *CsBPC1* to *CsBPC4*. They were classified into two groups based on phylogenetic analysis. Their predicted gene structures and conserved motifs were subsequently analyzed. Furthermore, we investigated their expression patterns in various tissues and in response to different stresses and plant hormones by qRT-PCR. To further verify the cellular functions of the *CsBPCs*, transgenic tobacco plants constitutively overexpressing *CsBPC2* were generated and seed germination experiments with different concentrations of salt, polyethylene glycol (PEG), and abscisic acid (ABA) were conducted. This work provides a basis for exploring the potential functions of BPC genes, especially in stress resistance. This will enrich the stress tolerance theory of plants and lay a theoretical foundation to alleviate the detrimental effects on cucumber growth caused by various abiotic stresses.

## 2. Results

### 2.1. Identification and Characterization of Cucumber BPC Genes

**Figure 1:**
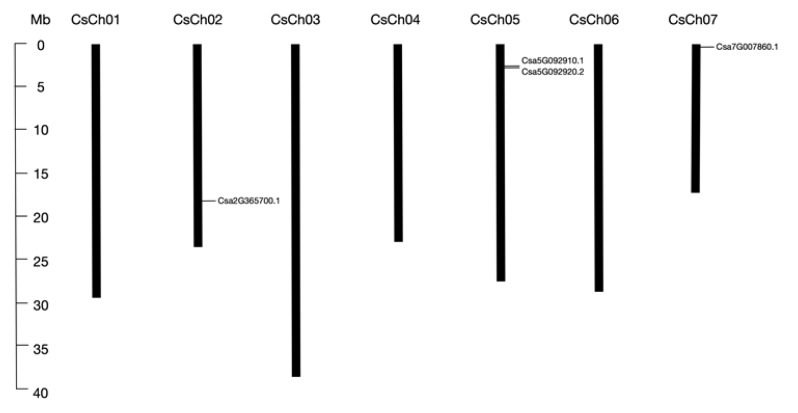
Localization of four cucumber BPC genes on chromosomes. There are seven chromosomes in cucumber, and the chromosome number is indicated at the top of each chromosome. The scale bar on the left side indicates the size of chromosome. The relative positions of four BPC genes are marked on the chromosomes.

To identify BPC family genes in cucumber, we performed BLASTP searches against the Cucumber Genome Database using seven *Arabidopsis* BPC proteins as query sequences, and confirmed the candidate sequences using the Pfam and SMART databases. We detected four BPC family proteins containing the GAGA binding-like domain (Table 1), which was consistent with our previous identification [21]. However, here we found that *Csa5G092920.2* and *Csa7G007860.1* had two and three transcripts, respectively, and we selected genes with longest encoding protein sequences for subsequent analysis. Two of the four members were distributed on chromosome five; the other two were distributed on chromosomes two and seven, respectively (Figure 1). All proteins shared similar parameters. The amino acid lengths of these four genes ranged from 279 to 338 aa, with molecular weights ranging from 31.1 to 37.8 kDa. The isoelectric points of all four BPC proteins were relatively high (pI > 9), indicating that they are rich in alkaline amino acids. Subcellular location prediction showed that all four members were localized to the nucleus.

### 2.2. Phylogenetic Gene Structure and Motif Analysis

**Figure 2:**
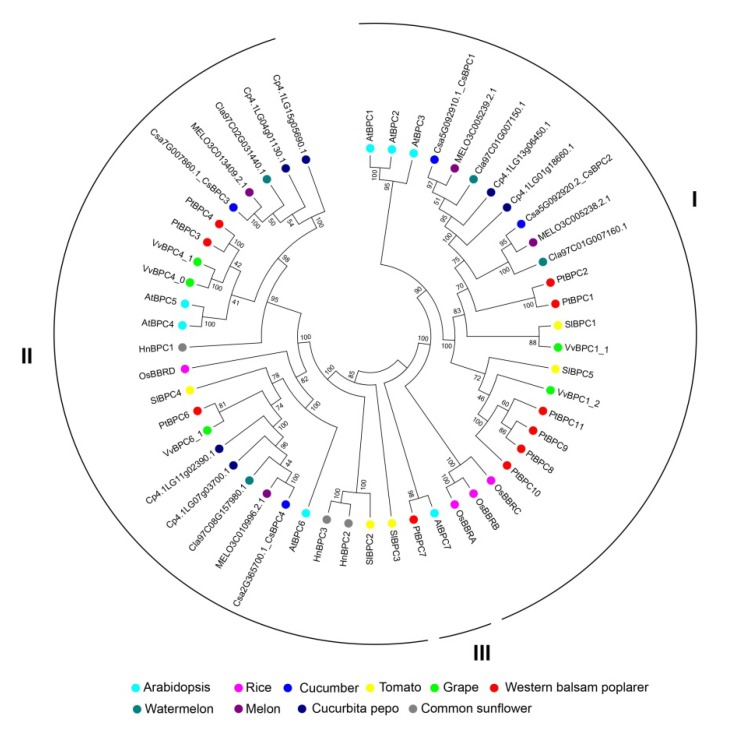
Phylogenetic analysis of BPC proteins from cucumber and other species. BPC polypeptide sequences generated from four cucumber, seven *Arabidopsis*, four rice, four watermelon, four melon, six cucurbita pepo, five grape, ten western balsam poplar, three common sunflower, and five tomato (SlBPCs) plants were used to construct an unrooted neighbor-joining phylogenetic tree by MEGA7 software with 100 bootstrap replicates. The different colored dots indicate different species.

**Figure 3:**
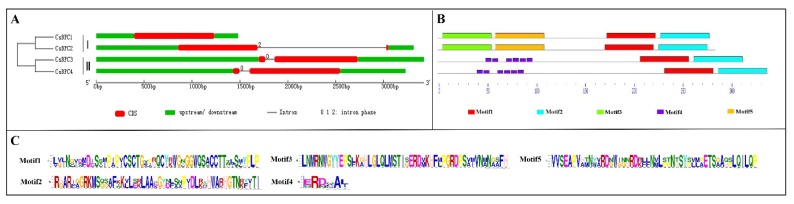
Gene structure and conserved motif analyses of cucumber BPC members. (**A**) Exon–intron organization of CsBPC genes. Red boxes represent exons and the black lines represent introns. The numbers 0, 1, and 2 represent the intron phases. The untranslated 5′- and 3′- regions (UTR) are shown with green boxes. (**B**) Conserved motif in cucumber BPC proteins. Five motifs with E-value < 0.05 are presented with different colored boxes. (**C**) The sequences of five identified motifs in cucumber BPC proteins.

To gain insight into the evolution of the *BPCs* in cucumber, a phylogenetic tree was constructed using BPC proteins from different plant species, including cucumber, watermelon, melon, cucurbita pepo, grape, western balsam poplar, common sunflower, *Arabidopsis*, rice and tomato. As shown in Figure 2, the 52 BPC proteins were classified into three groups, which was consistent with previous classification in *Arabidopsis* [10]. *Csa5G092910.1* and *Csa5G092920.2* belonged to group I; here, we named them *CsBPC1* and *CsBPC2*, respectively. *Csa7G007860.1* and *Csa2G365700.1* belonged to group II; here, we named them *CsBPC3* and *CsBPC4*, respectively. The numbers of BPC genes in group I, group II, and group III were 24, 26, and 2, respectively. BPC genes in *Arabidopsis* and western balsam poplar were clustered in each group, whereas, those in other eight species were just clustered in group I and group II. This indicated that BPC genes in group I and group II were relatively more conserved than those in group III, and that BPC genes in group III might be lost more easily during the evolutionary process. Besides, since cucumber, watermelon, melon, and cucurbita pepo belong to the Cucurbitaceae family, their evolutionary processes were more similar with each other than that with other species. Moreover, each cucumber BPC gene was closest with that in melon. Figure 3A illustrates the exon–intron organizations of the CsBPC genes. All the CsBPC genes possessed one intron, with the exception of *CsBPC1*, which lacked an intron. Additionally, the introns of *CsBPC3* and *CsBPC4* were phase zero introns, while *CsBPC2* had a phase two intron. The conserved domains of the CsBPC proteins were then analyzed and five conserved motifs were identified (Figure 3B). In general, members in the same group shared similar motif compositions, whereas members in different groups had differing motif compositions. Both group I members possessed four motifs, while both group II members had three motifs. Motifs one and two were found in all four members. Motifs three and five were only found in the group I members, and motif four only in the group II members. Furthermore, motif one contained five conserved cysteine residues (Figure 3C), which is the unique hallmark of the BPC transcription factor family [10,11].

### 2.3. Expression Profiles in Different Tissues

**Figure 4:**
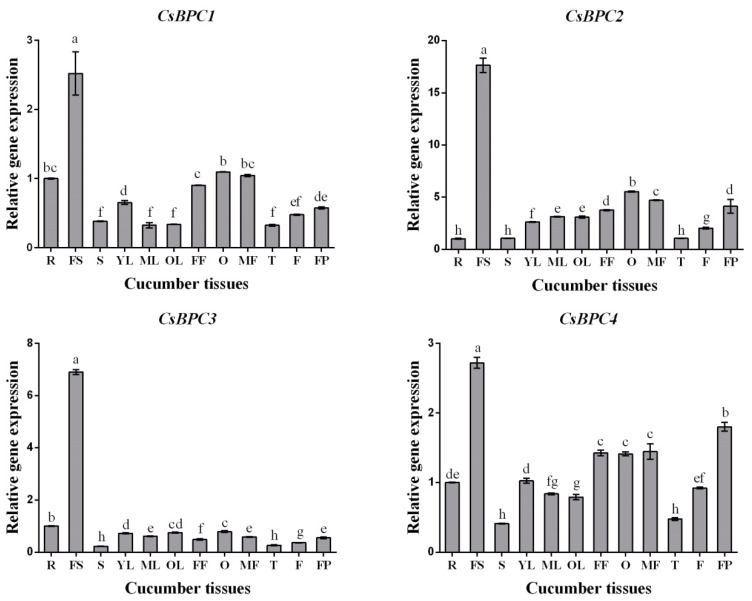
Expression analysis of CsBPC genes in different tissues. Transcript levels of *CsBPCs* were analyzed by qRT-PCR in roots (R), stems (S), top young leaves (YL), middle mature leaves (ML), basal old leaves (OL), blooming male flowers (MF), blooming female flowers with ovaries removed (FF), ovaries (O), tendrils (T), fruits (F), mature fruits’ seeds (FS), and pulps (FP). The expression levels of roots were defined as 1. Values are means ± SD (*n* = 3). Different letters indicate significant differences (*p* < 0.05).

To confirm the potential functions of the *CsBPCs* in cucumber growth and development, the expression patterns of the four *CsBPC* genes were analyzed by qRT-PCR in 12 different tissues. As shown in Figure 4, all four *CsBPC* genes were expressed to varying degrees in the tissues tested, and they shared the highest expression levels in seeds and the lowest levels in tendrils and stems. The expression levels of the *CsBPCs* in different floral organs, including male flowers (MF), female flowers with ovaries removed (FF), and ovaries (O), showed that *CsBPC1*, *CsBPC2,* and *CsBPC3* were highly expressed in ovaries compared with the other two organs, whereas *CsBPC4* was expressed at almost the same level in all three organs. Additionally, when comparing the expression levels in leaves at different developmental stages, we found that *CsBPC1* and *CsBPC4* had relatively higher transcription levels in young leaves than in mature and old leaves, which indicated that they may play an important role in the early stages of leaf growth and development. Collectively, the results indicated the *CsBPC* genes may play vital roles in many cellular processes in cucumber growth and development, and that the different members have overlapping but distinct functions. However, it is necessary to do plenty of complex experiments to investigate the functions of *CsBPCs* in regulating cucumber growth and development.

### 2.4. Expression Patterns of the CsBPC Genes under Different Abiotic Stress and Phytohormone Treatments

**Figure 5:**
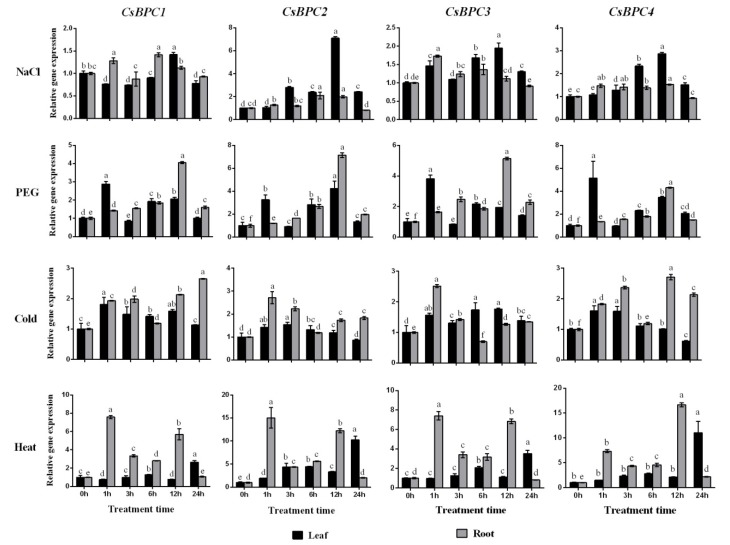
Expression patterns of CsBPC genes under different abiotic stress treatments. Cucumber seedlings at three-leaf-stage were exposed to 100 mM NaCl, 10% PEG 6000, cold (5 °C), and heat (38 °C) conditions, and qRT-PCR was performed to examine the transcript levels of *CsBPCs* in response to different abiotic stresses. The expression levels in non-stressed leaves and roots were set as 1. Values are means ± SD (*n* = 3). Different letters indicate significant differences (*p* < 0.05).

**Figure 6:**
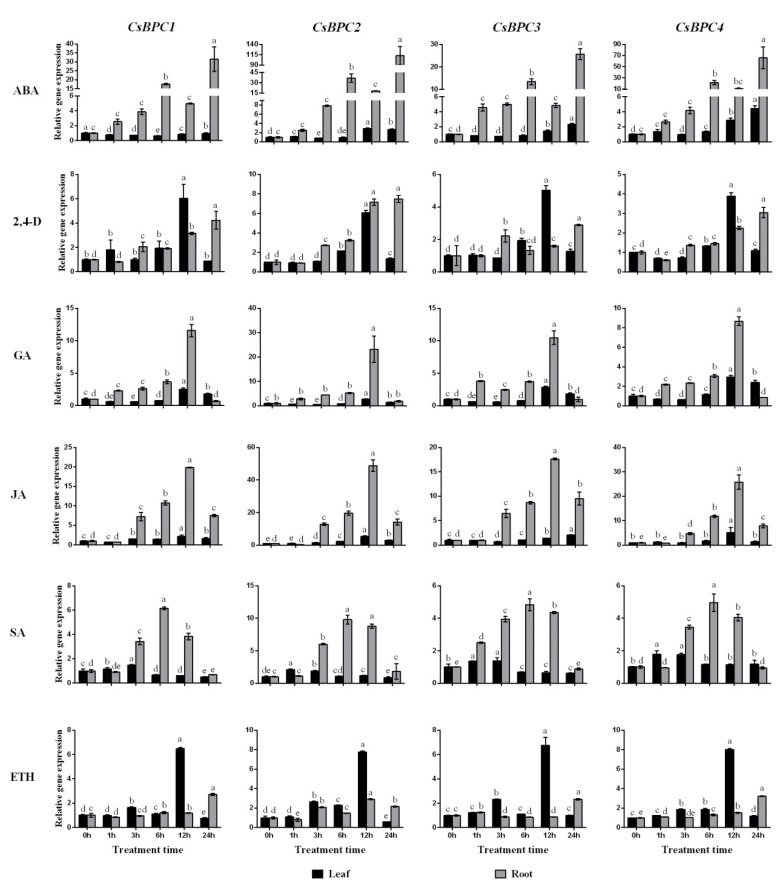
Expression patterns of CsBPC genes under different phytohormone treatments. Cucumber seedlings at three-leaf-stage were exposed to ABA, SA, JA, ETH, 2,4-D, and GA conditions, and qRT-PCR was performed to examine the transcript levels of *CsBPCs* in response to different phytohormones. The expression levels in non-stressed leaves and roots were set as 1. Values are means ± SD (*n* = 3). Different letters indicate significant differences (*p* < 0.05).

Increasing evidence suggests that BPC transcription factors play important roles in regulating plant growth and development. However, the involvement of *BPCs* in responses to abiotic stresses and phytohormones is not clear. To confirm the involvement of the *CsBPCs* in abiotic stress and hormone responses, their transcript abundances were investigated under NaCl, PEG, cold (5 °C), heat (38 °C), ABA, SA, JA, ETH, 2,4-D, and GA treatments. Overall, the expression of all four CsBPC genes was induced by all the treatments tested (Figure 5 and Figure 6). For instance, under the NaCl, PEG, and cold treatments, the expression levels of *CsBPC2* in roots or leaves increased by a maximum of 6.1-, 6.1-, and 1.7-fold, respectively, the most among the four genes. Under heat stress, the transcript level of *CsBPC4* was the highest at 12 h in roots (increased by 15.6-fold), followed by *CsBPC2* in roots (increased by 14.0-fold). In response to the application of exogenous hormones, including ABA, GA, JA, and SA, the transcript levels of all four CsBPC genes, especially *CsBPC2*, in roots were dramatically induced with prolonged treatment, whereas the transcript levels in leaves were increased slightly. Additionally, the application of ABA more significantly induced gene expression in roots compared with the other three hormones. Notably, the expression level of *CsBPC2* was increased by 112.1-fold after 24 h of ABA treatment. However, the expression patterns under ETH treatment were just the opposite, with higher gene expression induction in leaves than in roots. Lastly, application of 2,4-D highly induced the expression of all four genes in both roots and leaves, with maximal transcript levels at 12 h in leaves and 24 h in roots. Thus, the *CsBPCs* are involved in, and positively induced by, various abiotic stresses and phytohormones.

### 2.5. Germination Assays under Stress and ABA Treatments

Due to the fact that the above results showed that *CsBPC* expression was induced by various abiotic stresses and phytohormones, especially *CsBPC2*, whose expression level increased the most under the majority of treatments, a vector for constitutive overexpression of *CsBPC2* was constructed and transferred into tobacco plants to further study the cellular functions mediated by *CsBPC2* in response to environmental stresses and plant hormones. Genomic DNA of 10 independent T0 progeny transgenic tobacco lines (1, 2, 3, etc.) was extracted for PCR confirmation (Additional file 3). The results showed that *CsBPC2* was successfully integrated into the tobacco genome. Additionally, qRT-PCR indicated that *CsBPC2* was highly expressed in the transgenic lines, but no expression was observed in wild-type (WT) plants (Additional file 3). Thus, we chose two highly expressing lines, 5 and 6, for further experiments.

**Figure 7:**
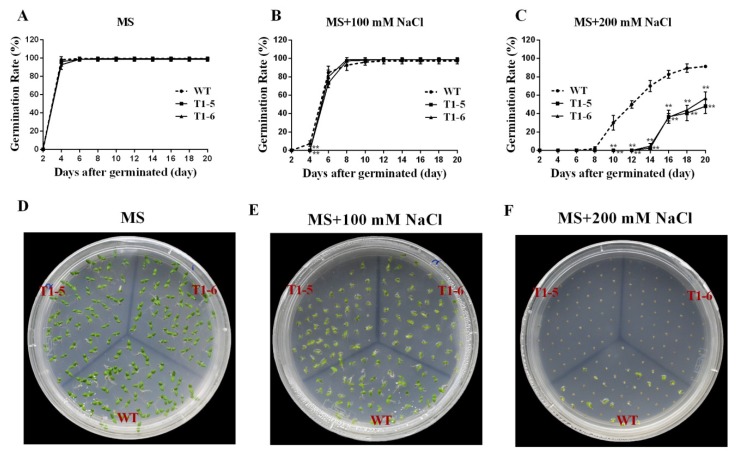
Effects of NaCl application on germination rates between wild-type (WT) and transgenic plants. Fifty seeds of WT and T1 transgenic plants were sown on sterile MS solid medium supplemented with different concentrations of NaCl (**A**–**C**). Values are means ± SD (*n* = 3). The photographs (**D**,**E**) were taken after being sown for 9 d, photograph **F** was taken after being sown for 13 d. * and ** Significant at *p* < 0.05 and *p* < 0.01 compared with WT, respectively.

To further verify the effects mediated by *CsBPC2* in abiotic stresses and plant hormone responses, seeds of WT and *CsBPC2*-overexpression plants (OE) were sown on MS solid medium containing different concentrations of NaCl, PEG, and ABA. As shown in Figure 7A,D, the germination times and rates of the WT and OE seeds displayed no difference when sown on MS medium. However, in the presence of 100 mM NaCl, the germination of OE was slightly inhibited compared with the WT (Figure 7B,E). Under 200 mM NaCl, the germination rates and times of all genotypes were significantly inhibited, but the transgenic lines were inhibited much more than the WT (Figure 7C,F). The seeds of T1-5 and T1-6 began to germinate at day 14, six days later than the WT, and their germination rates were just 2.67% and 4.67%, respectively, while that of the WT was 70%. On the last day of the treatment, the germination rates of T1-5 and T1-6 were 48.0% and 56.67%, respectively, which were significantly lower than that of the WT (91.33%).

**Figure 8:**
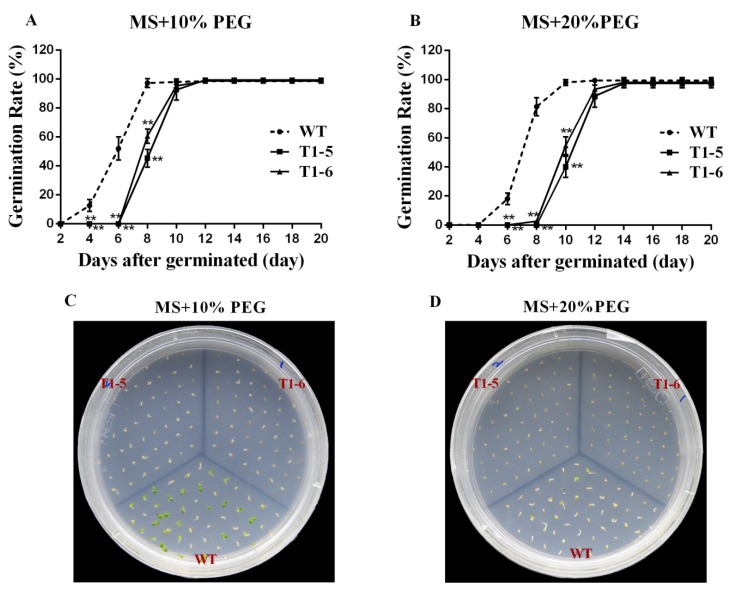
Effects of PEG application on germination rates between wild-type (WT) and transgenic plants. Fifty seeds of WT and T1 transgenic plants were sown on sterile MS solid medium supplemented with different concentrations of PEG (**A**,**B**). Values are means ± SD (*n* = 3). The photographs (**C**,**D**) were taken after being sown for 9 days. * and ** Significant at *p* < 0.05 and *p* < 0.01 compared with WT, respectively.

In the presence of 10% PEG, although there was no difference in the final germination rates of the WT and OE seeds, the T1-5 and T1-6 seeds started to germinate at day eight, four days later than the WT, and their germination rates were 45.33% and 60.67%, respectively, while that of the WT was 97.33% (Figure 8A,C). Under 20% PEG, the WT seeds started to germinate at day six, and the germination rate was 18.0%, while T1-5 and T1-6 seeds started to germinate at day 10 and day eight, respectively, and their germination rates were 40.0% and 2.67%, respectively; however, the final germination rates of all seeds displayed no difference (Figure 8B,D).

**Figure 9:**
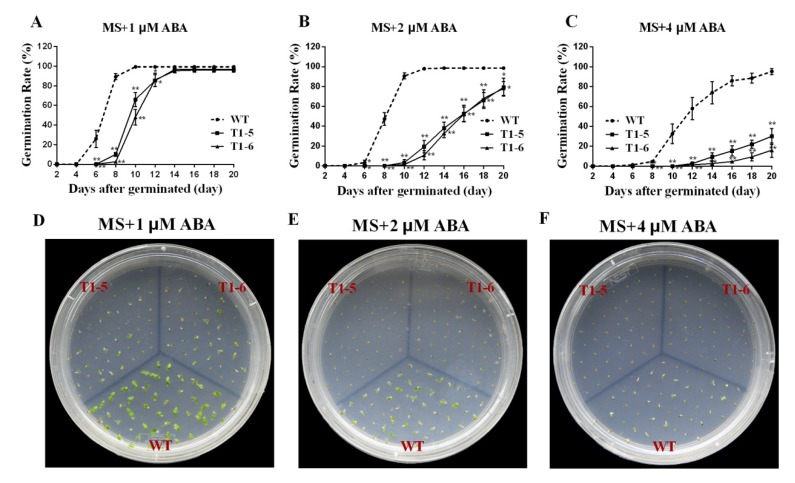
Effects of ABA application on germination rates between wild type (WT) and transgenic plants. Per 50 seeds of WT and T1 transgenic plants were sown on sterile MS solid medium supplemented with different concentrations of ABA (**A**–**C**). Values are means ± SD (*n* = 3). The photographs (**B**–**D**) were taken after being sown for 9 d. * and ** Significant at *p* < 0.05 and *p* < 0.01 compared with WT, respectively.

In the presence of 1 μM ABA, the OE plants displayed great inhibition of germination compared with the WT (Figure 9A,D). When the ABA concentration was increased to 2 μM, the inhibition was even greater (Figure 9B,E). The T1-5 and T1-6 seeds started to germinate at day 10, four days later than the WT, and their germination rates were 3.33% and 1.33%, respectively, while that of the WT was 90.67%. At the end of the treatment, the germination rates of T1-5 and T1-6 were 79.33% and 78.0%, respectively, which were significantly lower than that of the WT (98.67%). When the ABA concentration was increased to 4 μM, the final germination rates of T1-5 and T1-6 were just 30.0% and 16.0%, respectively, which were dramatically lower than that of WT (95.33%) (Figure 9C,F).

## 3. Discussion

Transcription factors play essential roles in regulating plant growth and development as well as responses to diverse abiotic stresses by activating or repressing related downstream genes [26]. With the completion of genome sequencing for more species, many major transcription factor families with large numbers of members have been identified and characterized in numerous plants, such as *bHLH* [27], *MYB* [28], and *WRKY* [29]. The functions of these transcription factors in growth, development, and stress responses have been studied extensively. However, no extensive studies have been done on the BPC family in recent years. Studies of BPC transcription factors have mainly focused on the regulation of plant growth and development [12,14,15,25], and almost no research has been reported on their regulation of stress resistance. Additionally, functional studies of the BPC family have primarily been done in the model plants *Arabidopsis* [12,14,15,25] and rice [22]; there is little information available about the BPC genes in other plant species.

In the present study, we identified and characterized four predicted BPC proteins in cucumber at the whole-genome level, and compared them with four watermelon, four melon, six cucurbita pepo, five grape, ten western balsam poplar, three common sunflower, seven *Arabidopsis*, four rice, and five tomato BPC proteins. Phylogenetic analysis classified these 52 BPC proteins into three groups (Figure 2), the same as in *Arabidopsis* [10]. Additionally, group I and group II contained similar members, whereas group III just contained two members, indicating that the BPC genes had diversified before various species diverged and that members in group Ⅲ might be lost during the evolutionary process, however, the functions of these genes remain unclear. Here, the four CsBPC genes were classified into group I and group II, with each group containing two members. Gene structure (Figure 3A) and conserved motif (Figure 3B) analyses indicated that the CsBPC genes in the same group shared similar exon–intron and motif organizations, whereas members in different groups had differing exon–intron and motif compositions, which may suggest a closer evolutionary relationship between members of the same group and functional diversification among the members of different groups. Moreover, the two BPC groups showed a distinct difference in their N-terminal structural domains, which have been predicted to form zipper-like coiled-coil structures and may function as dimerization domains, protein–protein interactions domains, or nuclear and nucleolar localization signals [9,10,30]. Conversely, all four CsBPC members shared a highly conserved domain at their C-terminus that is important for DNA binding. The hallmark of this domain is the existence of five cysteine residues with defined positions and spacing [9,10,30]. These cysteines were previously believed to form a zinc finger-like structure for direct recognition of GAGA motifs [10]. However, the results of Theune et al.’s [31] research contradicted the suggestion of a zinc finger-like DNA-binding mechanism. Instead, they proposed that the conserved cysteines form inter- and intramolecular disulfide bonds to stabilize a parallel conformation of monomers, and that this conformation is required for neighboring GAGA motif recognition and binding.

Tissue-specific expression analysis is usually performed to predict the biological functions of genes in plant growth and development. Therefore, we investigated the expression of the four CsBPC genes by qRT-PCR in 12 different tissues. The results revealed that all four cucumber BPC genes were ubiquitously expressed in all the tissues tested, and that they shared the highest expression levels in seeds and the lowest levels in tendrils and stems (Figure 4), which may suggest a vital role for the *CsBPCs* in cucumber growth and development processes and, particularly, in seed development. Taking these results together, BPC transcription factors are essential for maintaining a wide range of normal growth and developmental processes, but further studies are needed to determine the functions of *CsBPCs* in regulating cucumber growth and development. 

As there were few studies on *BPCs* in response to abiotic stresses and phytohormones, we did the expression analysis of *CsBPCs* under various stresses and hormones treatments. qRT-PCR showed that the transcriptional levels of all four CsBPC genes were induced by various abiotic stress treatments (NaCl, PEG, cold, and heat) (Figure 5) and hormone treatments (ABA, SA, JA, ETH, 2,4-D, and GA) (Figure 6), indicating that abiotic stresses and hormones might be activators of the *CsBPCs*.

To further research the cellular functions of the *CsBPCs*, especially in plant responses to hyperosmotic stress, transgenic tobacco plants constitutively overexpressing *CsBPC2*, whose expression level increased the most under the majority of abiotic stress and hormone treatments, were generated. No apparent differences in seed germination rates or seedling size were observed when seeds of the WT and transgenic lines were germinated under normal growth conditions (Figure 7). However, when seeds of these plants were germinated on MS solid medium supplemented with different concentrations of NaCl (Figure 7) and PEG (Figure 8), the germination rates and times of the transgenic lines were significantly inhibited compared with those of the WT. Additionally, when seeds were germinated on MS solid medium supplemented with different concentrations of ABA, the inhibition was even more severe (Figure 9). Collectively, these results indicated that *CsBPC2* plays a negative role in modulating seed germination under hyperosmotic conditions. *CsBPC2* may also function in ABA-inhibited seed germination and hypersensitivity to ABA-mediated responses. The altered stress responses exhibited by *CsBPC2* transgenic seeds may be caused by changes in phytohormones levels. Numerous studies have demonstrated that plant hormones are critical for regulation of seed dormancy and germination [32,33]. For example, the plant hormones gibberellin [34,35], cytokinin [36], brassinosteroids [37], and ethylene [38] are positive regulators of seed germination, whereas abscisic acid is a negative regulator of seed germination [39,40]. A previous study confirmed a role for *BPCs* in the regulation of the cytokinin content in the meristem [14]. In *Arabidopsis bpc1-1 bpc2 bpc3* triple mutants, the expression levels of both *ISOPENTENYLTRANSFERASE 7* (*IPT7*) and *ARABIDOPSIS RESPONSE REGULATOR 7* (*ARR7*), two genes involved in cytokinin biosynthesis and responsiveness, respectively [41,42,43], were upregulated, resulting in an increase in the cytokinin concentration in inflorescence meristems. Moreover, *Arabidopsis bpc1-1 bpc2 bpc4 bpc6* quadruple mutants showed reduced sensitivity to ethylene responses, the lack of an apical hook, and delayed senescence [12]. The hormone-mediated regulatory mechanisms may be complex or there may be other signal perception or transduction pathways involved in seed germination regulation under hyperosmotic conditions; we need to research these issues in future studies. Additionally, further studies are needed to test the function of *CsBPC2* at the seedling stage in responses to abiotic stresses as genes can participate in different regulatory mechanisms at different growth stages and, thus, exhibit different functions. Understanding these matters will aid in understanding the *BPC*-mediated signaling cascades in plant resistance to stresses.

## 4. Materials and Methods

### 4.1. Identification of BPC Gene Family Members in Cucumber

Seven *Arabidopsis* BPC proteins sequences were downloaded from TAIR (http://www.arabidopsis.org) and the obtained sequences were used as queries to identify all GAGA-binding transcriptional activators in cucumber by searching against the Cucumber Genome Database (http://cucurbitgenomics.org/organism/2) with the BLASTP program and default E-value. All predicted cucumber BPC proteins were confirmed using the Pfam (http://pfam.xfam.org) [44] and SMART (http://smart.embl-heidelberg.de/) databases [45]. The amino acid lengths, chromosome locations, and strand directions of the BPC genes were also obtained from the databases. Physicochemical parameters, including the isoelectric point (pI) and molecular weight (kDa), were calculated using the compute pI/Mw tool in ExPASy (http://web.expasy.org/compute_pi/). Subcellular location prediction was performed using the software CELLO 2.5 (http://cello.life.nctu.edu.tw/) [46]. The information on chromosome locations of the four BPC genes were drawn using MapInspect tool.

### 4.2. Phylogenetic Gene Structure and Conserved Motif Analyses

The sequences of the four cucumber, four watermelon, four melon, six cucurbita pepo, and seven *Arabidopsis* BPC genes were downloaded from the Cucumber (Chinese Long) v2 Genome Database (http://cucurbitgenomics.org/organism/2), Watermelon (97103) v2 Genome Database (http://cucurbitgenomics.org/organism/21), Melon (DHL92) v3.6.1 Genome Database (http://cucurbitgenomics.org/organism/18), Cucurbita pepo (Zucchini) Genome Database (http://cucurbitgenomics.org/organism/14), and TAIR (https://www.arabidopsis.org/), respectively. The sequences of four rice, five grape, ten western balsam poplar, three common sunflower, and five tomato BPC proteins were obtained from UniProt (https://www.uniprot.org/). All full-length protein sequences of cucumber, *Arabidopsis*, tomato, watermelon, melon, cucurbita pepo, grape, western balsam poplar, common sunflower, and rice were aligned using the Clustal W program with default parameters, and then an unrooted neighbor-joining phylogenetic tree was constructed using the MEGA7 software with 100 bootstrap replicates [47]. The exon–intron compositions of the cucumber BPC genes were predicted by comparing the CDS sequences with their corresponding genomic sequences using the online program GSDS 2.0 (http://gsds.cbi.pku.edu.cn/) [48]. The conserved motifs of the cucumber BPC proteins were identified using the online tool MEME (http://meme-suite.org/tools/meme) [49] with the following parameters: any number of repetitions site distribution, 5 motifs found, and minimum and maximum motif widths—6 and 50, respectively.

### 4.3. Plant Material, Growth Conditions, and Treatments

Cucumber (*Cucumis sativus* L. cv. ‘changchunmici’) seeds (saved by our laboratory) were germinated on moist gauze in an incubator at 28 °C under dark conditions. The germinated seeds were sown in plastic plugs (32 holes) filled with nursery substrate (peat: vermiculite: perlite = 2:1:1) in a climate chamber at The Institute of Vegetables and Flowers, Chinese Academy of Agricultural Sciences. When the seedlings were at the two-leaf-stage, they were transplanted into a greenhouse. When their fruits became ripe, samples of various tissues were collected, including fresh roots (R) and stems (S), top young leaves (YL), middle mature leaves (ML), basal old leaves (OL), blooming male flowers (MF), blooming female flowers with ovaries removed (FF), ovaries (O), tendrils (T), around 10-day-old fruits (F), and seeds (FS) and pulp (FP) gathered from the mature fruits. All samples were immediately frozen in liquid nitrogen and stored at −80 °C for RNA extraction and tissue expression analysis.

For different stress and phytohormone treatments, batches of five seedlings at the one-leaf-stage were transferred to a 5 L (33 cm × 25 cm × 11 cm) plastic tank filled with an aerated complete nutrient solution (pH 6.0–6.5) containing: Ca(NO_3_)_2_·4H_2_O 4 mM, KNO_3_ 6 mM, MgSO_4_·7H_2_O 2 mM, NH_4_H_2_PO_4_ 1 mM, EDTA-FeNa 80 µM, H_3_BO_3_ 46.3 µM, MnSO_4_·H_2_O 9.5 µM, ZnSO_4_·7H_2_O 0.8 µM, CuSO_4_·5H_2_O 0.3 µM, and (NH_4_)_6_Mo_7_O_2_·4H_2_O 0.02 µM. The experiment was carried out under normal conditions (28 °C/14 h light, 18 °C/10 h dark) in a climate chamber. When the cucumber seedlings were at the three-leaf-stage, 100 mM NaCl, 10% polyethylene glycol (PEG) 6000, 100 μmol ABA, 100 μmol salicylic acid (SA), 100 μmol jasmonic acid (JA), 50 μmol ethephon (ETH), 50 μmol 2,4-dichlorophenoxyacetic acid (2,4-D), and 50 μmol gibberellin (GA) were added to the nutrient solution. For cold and heat treatments, cucumber seedlings were cultivated at 5 °C and 38 °C, respectively. Samples of cucumber seedlings were taken after 0, 1, 3, 6, 12, and 24 h of the different treatments for gene expression analysis.

### 4.4. Vector Construction and Tobacco Transformation

Two primers were designed to amplify the *CsBPC2* gene based on its CDS sequence (Additional file 2. S2). Then, the *CsBPC2* CDS was inserted into the *Bam*HI/*Pml*I restriction sites downstream of the 35S promoter of the vector 1305 (35S:*CsBPC2*) using In-Fusion technology. The construct was introduced into the *Agrobacterium tumefaciens* strain EHA105 for tobacco (*Nicotiana tabacum* cv. NC89) transformation using the leaf disc method [50]. Tobacco genomic DNA from young leaves was extracted for PCR confirmation using primers for *Hyg* (Additional file 2. S3) and *CsBPC2* CDS amplification to determine whether *CsBPC2* was integrated into the tobacco genome. Additionally, qRT-PCR was performed to further test the expression of *CsBPC2* in the transgenic tobacco plants using primers for *CsBPC2*, which is unique to cucumber (Additional file 2. S4).

### 4.5. RNA Extraction and qRT-PCR Analysis

Total RNA was extracted using an RNA prep pure Plant Kit (TANGEN) and first-strand cDNA was synthesized using a PrimeScript™ RT reagent Kit with gDNA Eraser (Perfect Real Time) (TaKaRa) according to the manufacturer’s instructions. Quantitative RT-PCR was performed following the instructions of the SYBR^®^ Premix Ex Taq™ Kit (TaKaRa) on a Mx3000P real-time PCR instrument (Agilent). The experiments were performed with three biological replicates, each with three plants. Relative gene expression was calculated using the 2^−△△Ct^ method [51]. The *CsBPC* specific primers used for expression pattern analysis in different tissues and under abiotic stress and hormone treatments are shown in Additional file 2. S1.

### 4.6. Seed Germination of Transgenic Tobacco Plants under Hyperosmotic Stress Conditions

Seeds of the wild-type (WT) and T1 progeny obtained from transgenic tobacco plants were germinated on sterile MS solid medium containing 30 g/L sucrose. To test the effects of hyperosmotic stress or phytohormones on germination, 100 and 200 mM NaCl, 10% and 20% PEG-6000, and 1, 2, and 4 μM ABA were added to the medium. Germination rate assays were carried out on three replicates of 50 seeds. The seeds were geminated in an incubator (26 °C/14 h light, 10 h dark) for 20 days.

### 4.7. Statistical Analyses

Values presented are means ± standard deviation (SD) of three replicates. Statistical analyses were carried out by an analysis of variance (ANOVA) using SAS (SAS Institute, Cary, NC, USA) software.

## 5. Conclusions

The ubiquitous expression of the four *CsBPCs* in various tissues perhaps to some extent implied they have crucial roles in regulating cucumber growth and development. Moreover, analysis of *CsBPC* expression under various abiotic stress (NaCl, PEG, cold, and heat) and hormone (ABA, SA, JA, ETH, 2,4-D, and GA) treatments revealed that the *CsBPCs* respond to phytohormones and abiotic stresses. Additionally, tobacco plants overexpressing *CsBPC2* showed an inhibited germination phenotype when treated with NaCl, PEG, and ABA, compared with the wild type. This further confirmed the regulatory function of the *CsBPCs* in stress and hormone responses. The present results reveal the potential roles of *CsBPCs* in cucumber development and abiotic stresses responses, and provide a basis for further functional studies of the BPC genes in cucumber and other plant species.

## Figures and Tables

**Table 1:** Characteristics of the BASIC PENTACYSTEINE (BPC) family in cucumber. Gene ID with black bold means more than one transcripts.

Gene ID	Length (aa)	Molecular Weight (KD)	Chromosome	Location	pI	Strand Direction	Subcellular Location
Csa2G365700.1	338	37.8	2	17669793–17673023	9.52	+	Nuclear
Csa5G092910.1	279	31.1	5	2693816–2695298	9.62	-	Nuclear
**Csa5G092920.2**	284	31.7	5	2696954–2700269	9.84	-	Nuclear
**Csa7G007860.1**	313	35	7	388479–391899	9.6	+	Nuclear

## Supplementary Materials

Supplementary materials can be found at https://www.mdpi.com/1422-0067/20/20/5048/s1. Additional file 1: Sequences of CsBPC genes. A: The coding sequences of *CsBPCs*. B: The protein sequences of *CsBPCs*. C: The 2 kb genomic DNA sequences upstream of the initiation codon. Additional file 2: Sequences of the primers used in this study. Additional file 3: Confirmation of transgenic tobacco plants by PCR and qRT-PCR methods.

## Abbreviations

wt	wild-type
qRT-PCR	quantitative real-time polymerase chain reaction
PEG	polyethylene glycol
MS	Murashige and Skoog
ABA	abscisic acid
