# Supplementary material for: Genome-Wide Identification and Characterization of Cucumber BPC Transcription Factors and Their Responses to Abiotic Stresses and Exogenous Phytohormones
Source: Int J Mol Sci. 2019 Oct 11;20(20):5048. doi: 10.3390/ijms20205048 (PMC6829308; doi:10.3390/ijms20205048)
Supplement: Supplementary file 1 [file ijms-20-05048-s001.zip › Additional file 1.pdf]

A:Coding sequences of CsBPC genes.

>Csa5G092910.1
ATGGATGACGATGCGTTAAACATGCGTAATTGGGGTTATTATGAGCCGTC
CTTCAAAGGGCATCTCGGCCTGCAGCTCATGTCCACCATTTCTGAGCGGG
ACATGAAACATTTCTTACCAGGCCGTGATCCTTCTGTTATGGTTAATGCCA
ATGGTTCCTTCCATCCTCGGGATTGCGTTGTTTCGGAAGCACCAGTGCATA
TGAACTATGTGAGGGACAATTGGGGGGGTAACAGAGATAGGTTTCTTAAT
ATGTTACCTACCAATCACAGCTATCCTGTTATGCCAGAAACTTCAGGAGCT
CACTCCTTGCAGATCTTGCAACCACCCTCTTCGTCGAGGGATGAAATAGC
AGCAAGTAGAGTTGAAGAGCCTCCAGTGAAGAAGGAAGGTGGGAAAGCA
AAGAAAAGACAGAGTAGTGAGGCTGGCCCCAAAGCCCCCAAAGCTAAAA
AACCGAGGAAACCAAAAGATACTAGCACGGCTGTTCAGCGTGTGAAACC
ACCAAAGAAGAACATTGATCTTGTTATAAATGGGATTGATATGGACATCT
CATGTATTCCAATCCCGGTTTGCTCTTGCACTGGAGCTCCTCATCAATGTT
ACAGGTGGGGATGTGGCGGTTGGCAATCTGCTTGTTGTACTACCAACATA
TCAACTTATCCTTTGCCCATGAGTGACAAAAGACGTGGGGCGAGGATAGC
TGGGCGAAAAATGAGTCAGGGTGCGTTTAAGAAGGTACTTGAAAAACTAG
CAGCTGATGGCTATAATTTTGCTAACCCAATCGATTTGAGGACTCACTGGG
CGAGACATGGTACGAATAAGTTTGTCACAATCAGGTAG

>Csa5G092920.2
ATGGATGGTGATGCTTTGAACATGCGTAATTGGGGTTACTATGAACCATCT
TTGAAAGCGCATCTTGGACTGCAGCTCATGTCCACAATTGGTGAGCGAGA
TGTGAAACATTTTATGCCTGGACGGGACCCGTCAGCTATTGTTAACATGA
ATGCCGCATTTCATCCACGGGAGTCTGTTGTTTCTGAAGCACCGGTAGCGA
CGAACTGGGCGAGGGATGGTTGGATAAATCATAGGGACAAGCTTTTCAAT
GTGTTATCCCCCAATACTAGTTATTCTCTGCTTGCAGAGACGTCAGCGGCA
CAACCTTTGCAAATTTTACAGCCACTTGACACATCAAGAGATGAAATGGT
TCTCAAGATTGAAGAACCACCTGTGAAGAAGGGAACTAAACAACCAAAG
AAACGACAGAATGGAGGTGCTCCCAAAACTCCGAAACCAAAGAAGCCTC
GAAAGCCTAAAAATAATGATCCTTCGGTTCAACAGGTGAAGGCACCAAAA
AAGAAGATGGAGCTTGTTATAAATGGGTTTGACATGGATATATCTAGTAT
CCCAATTCCAGTATGTTCTTGCACTGGAACTCCTCACCAATGTTATAGATG
GGGCTATGGTGGCTGGCAATCAGCTTGTTGTACCACAAGTTTATCTCTACA
TCCTTTGCCAATGAGTGAGAAGCGGCGAGGTGCAAGAATTGCTGGTCGAA
AAATGAGTCAAGGTGCTTTTAAGAAGGTTTTGGAGAAACTAGCAGCTCAA
GGCTATAACTTTTCTAACCCAATTGATTTAAGAAGCCATTGGGCAAGGCA
TGGGACCAATAAGTTTGTCACAATCAGGTGTACTAATCCTGTCTCAAATTA
A

>Csa7G007860.1
ATGGATGATGGCCGTCAACATGAAAACGGGAGGCACAAACTAGATTATTT
CCGAGGGAGCTCCTCACCATGGAATATGGTGCCCCCAAATCATGTAAAGG
AACCAAATGCCTTGGTCATGAATAAGAAGATCATGTCCATTATAGCCGAG
AGAGATGCTGCTATTCGGGAACGGAATTTAGCACTATCTGAGAAGAAGGA
GGCATTGGCTGCACGAGATGAGGCTCTCCGGCAGCGTGACGAGGCACTTG

TACAGCGTGATTCTGCGTTAATGGAAAGGGACAATGCCCTTGCTGCCCTT
GAAATTCGTGACAATGCTTCTAACTTCCCTCTTGGTGGTGGGGTCCAACGC
AAAACAAAGCGATTACACCATTTATCTAATCATATGCCGAACATATCTGA
AACTTCCTATGGTACAAAAGACGTGCATATAACTGATGCCTTTCCAATTAC
AGTTATAGCTTCTGAAGCTGTTAAGTCTCAGCAGGGAAAGCGAGCGAAGG
ATAACAAGTTAGTTTCATCAAAGACATCAAGGCCGCCAAGGAAGAAGGTT
GGCGAAGATTTGAATAGACATGCTGCCACCGATGGCACAAAATACAGAA
CTGATTGGGATGGTCAGGACGTGGGTTTGAATCTTGTAAGTTTTGATGATT
CTTCTATGCCAGCACCAATTTGTTCGTGTACTGGGTTCGCAAGACAGTGCT
ACAAATGGGGAAATGGGGGTTGGCAATCGTCTTGTTGTACCACCCATATG
TCCATGTATCCACTTCCACATTTGGAAAACAAACGCCATGCTCGTATGGGC
GGGCGGAAAATGAGTGGAAGTGTTTTACAAAATTGCTTAGTCGGCTAGC
AGCAGCAGGTCATGATCTGTCGGTACCAGTGGATCTTAAGGACCACTGGG
CAAGACACGGTACAAATCGCTACATAACAATCAGGTAG
>Csa2G365700.1
ATGGATGACAGTGGACACCGTGAGAATGGAAGGCACAAACCAGATCAGT
ATAAGTCAGCTCAGGGTCAGTGGATGATGCAGCATCAGCCCTCAATGAAG
CAGATAATGGCAATTATGGCTGAAAGAGACGCAGCCATTCAAGAAAGAA
ATTTGGCCCTCTCGGAGAAAAAGGCTGCACTGGCAGAGCGAGACATGGCA
TATCTACAGCGAGACGCTGCAATTGCAGAGAGGAACAATGCCCTTTTGGA
ACGAGACAACGCCATTGCTACTCTTCAGTATCGTGAAAACTCCATAAATA
ACAATTTATCATGTCCACCAGGATGCCAAATTGCTAGGGGAGTGAAGCAT
ATACATCATCCACAGCAGCAACACACACATCATGTGCCTCACATGAATGA
GAATAATTACAATTCAAGAGAAATGCTTGCTTCCAACGACCCTTGCCCAA
CATCCCCTGTCGCTTCTGAATCAACAAAGGCACGACGAAACAAGCGTCCA
AAAGAGGGAAAGACAGTCCCAACACCAAACAAGAAAGTTTCAAAAGGTC
CACGGAAGGTCAAAAGGGAGGCTGAAGACTTGAACAAAATAATGTTGGG
GAAGTCACAAGAATGGAAGGATGGAATTGGTATAATGAGTGCAGGTGAC
GATCTTAATAAACAGTTGGTAGTATCAAAATCAGATTGGAAAGGCCAGGA
TTTAGGATTAAACCAGGTTGCATTTGACGAATCAACCATGCCAGCTCCTAT
ATGCTCCTGCACAGGAGTAATAAGACAATGCTACAAATGGGGGAATGGTG
GATGGCAATCTGCATGTTGTACTACCACCCTCTCAATGTATCCATTACCTG
CCGTTCCCAACAAACGACATGCTCGACTTGGCGGTCGGAAAATGAGCGGA
AGTGCTTTTAACAAACTGCTTAGCCGCCTTGCAGCCGAAGGCCACGACCT
ATCCGCTCCAGTTGATCTTAAAAATCACTGGGCAAAGCATGGAACAAATC
GTTACATCACCATCAAGTAG

B:The protein sequences of CsBPC genes

>Csa5G092910.1
MDDDALNMRNWGYYEPSFKGHLGLQLMSTISERDMKHFLPGRDPSVMVNA
NGSFHPRDCVVSEAPVHMNYVRDNWGGNRDRFLNMLPTNHSYPVMPETSG
AHSLQILQPPSSSRDEIAASRVEEPPVKKEGGKAKKRQSSEAGPKAPKAKKPR
KPKDTSTAVQRVKPPKKNIDLVINGIDMDISCIPIPVCSCTGAPHQCYRWGCG
GWQSACCTTNISTYPLPMSDKRRGARIAGRKMSQGAFKKVLEKLAADGYNF
ANPIDLRTHWARHGTNKFVTIR

>Csa5G092920.2
MDGDALNMRNWGYYEPSLKAHLGLQLMSTIGERDVKHFMPGRDPSAIVNM
NAAFHPRESVVSEAPVATNWARDGWINHRDKLFNVLSPNTSYSLLAETSAAQ
PLQILQPLDTSRDEMVLKIEEPPVKKGTKQPKKRQNGGAPKTPKPKKPRKPKN
NDPSVQQVKAPKKKMELVINGFDMDISSIPIPVCSCTGTPHQCYRWGYGGWQ
SACCTTSLSLHPLPMSEKRRGARIAGRKMSQGAFKKVLEKLAAQGYNFSNPI
DLRSHWARHGTNKFVTIRCTNPVSN
>Csa7G007860.1
MDDGRQHENGRHKLDYFRGSSSPWNMVPPNHVKEPNALVMNKKIMSIIAER
DAAIRERNLALSEKKEALAARDEALRQRDEALVQRDSALMERDNALAALEIR
DNASNFPLGGGVQRKTKRLHHLSNHMPNISETSYGTKDVHITDAFPITVIASE
AVKSQQGKRAKDNKLVSSKTSRPPRKKVGEDLNRHAATDGTKYRTDWDGQ
DVGLNLVSFDDSSMPAPICSCTGFARQCYKWGNGGWQSSCCTTHMSMYPLP
HLENKRHARMGGRKMSGSVFTKLLSRLAAAGHDLSVPVDLKDHWARHGTN
RYITIR
>Csa2G365700.1
MDDSGHRENGRHKPDQYKSAQGQWMMQHQPSMKQIMAIMAERDAAIQER
NLALSEKKAALAERDMAYLQRDAAIAERNNALLERDNAIATLQYRENSINNN
LSCPPGCQIARGVKHIHHPQQQHTHHVPHMNENNYNSREMLASNDPCPTSPV
ASESTKARRNKRPKEGKTVPTPNKKVSKGPRKVKREAEDLNKIMLGKSQEW
KDGIGIMSAGDDLNKQLVVSKSDWKGQDLGLNQVAFDESTMPAPICSCTGVI
RQCYKWGNGGWQSACCTTTLSMYPLPAVPNKRHARLGGRKMSGSAFNKLL
SRLAAEGHDLSAPVDLKNHWAKHGTNRYITIK
C:The 2 kb genomic DNA sequences upstream of the initiation codon.
>Csa5G092910.1
CAGAACTACATACCAAACATAACAACAATAAGAGAAAGATTCTCAGGTTT
TGAATCTTCCCAATAACAGTACTTGGATGGCGATTGTTCAACCACATCATC
ATAATGGCTTGGAAATGAAAACAACAAAATATCCCAAGACGAGAAACGA
AATGGAAAAAAAAAAACAACAGAACAATCAAAGCGCAACACAAACCTTA
CAAGAGATGGGGAACACAATCAACAACAAAATTTTGCAACTAGACGCTA
ATTGTGTAGATCGACGTTCTTTCTAGATATTAATTTTAACGATAAAAACAA
GATTCCAGATCCAACATTTAATTCAATCAATCAAATAAATTAGGGTACAG
ATCTCGTTTCCGCAGTCCACAACTTGACATATTCAAATAATACGCAAAAA
AAAAAGAAAGAAAGAAAAGAAATCATCAAAATAAAAACATGTTCACATT
GGCAACATCGAAATGAAAACAGAAGCCCTAAACAGAAATCCCCAAAAGT
CAAAAACCCCTCAAAACATCAGATCTCCGACACCCATCATGCAGAAAGTG
CAAAAGATTAGGGTTTCCACAACATCCAAATCTGAAAAACCACAGATCTA
AGCAAAATTGAAGATAAAAAATCGGAGAATCGGAAAAACAAAAGAAAG
AAGGAAATGCATATTTATATGGGGATGCAGATAGTACAAGAAGGACCCAT
ATATTCAAACTCGAGAGAGAGAAAGGGAGAGAGAAAGGGAAGAAGAGA
GAGAAAGAGAAAGAAGGGGTAAGAGAGAGAGGACTTACTTGGGTGGGTG
TAAACGGCTCTTTAGGGTTTTGCCCTTCTGGAGAGAGAGAGACAGAGACA
GAGAGGAGAGAGGAGAGAGGAGAGAGGAGAGAGGAGAGAGGAGAGAG
GAGAGAGGAGAGAGGAGAGAGGAGAGAGGAGAGAGGAGAGAGGAGAG

AGGAGAGAGAAACCTGAGAGAGAGAGAGAGAGAGAGAGCTAAAAATGG
GTATCTATCAAAGAAGTAGTTGTCGCTTTAAAATAAGGAGAGAGGGAAAG
GGCAAACCAAAGCTATTCTATAAATTTCTTTCCTTTTTTCTTTTCTTTGTTA
ACTCTTTTTCTTTTTCTTTTTCTTTCTTTCTTTTTTTTTTCTTTTTAACCCCCA
AAACAAATTTATTATATTATATAATTTATATACCATGTTTTCATGTTTTCAT
GTTTTGGAGTAGGCGAAATTTTCCACATCACACAACCCTATCTTTATAATT
ATAATTATAATTATAATGCTTATTATTTATTTCTTCTTATTCTTATAATCTT
ATTCTTTTGAGTTTTTGAGGGCTCCACATTCCATTTCTCTTCTTTCCCTTTTC
TATTTATTCTATTGTCTAATCTATTCTTATCTACTTTCTCCTCTTCTTCATTC
TATTCTTCCTCTTTCTCATTATCAACTCTAATTTTGTTTCTCCCTATTATATT
ATTTCCTTCTCTTTTCTTTTCTCCTTTCTACTTCACTTTTCTTCCCAATCATC
CTTTCCATTTAATCACATTCCATCACACTTTTTTCAGTTTCTAATTATTTCA
TTTCTCAACTTTTACCATTTTCTAAATTTTCAACATAAATAAACACATAGTT
GTAAATACAATAACATAATGTACACAAATTTGTAAATATAGAATAAAGAG
ACTATCAATGATACACTATAGAGCGTTAGTAGAGTTTTTTTTGTAACTTTG
AAACACCAAATCACATTTGGCATAGATGAAGTACCTTAGCGATGTTAATC
CAATAGCCAAGAGCTTTAAATATAAGAAGGGAAAAGAGATTGATCTGAC
CTCTAATTTTGTAATATTGATATCATAGTAATGCGATTTCAATTATTAGAT
GGTAGATAGGATTCTCTTCATATAATCTTTGGTCTACAAATAAGCTGGTCC
GTCTCTTTAGTAATAATGGACCTGTAGTGGGGTATAATATAGACTAATTAT
CAGACTAATATGATTTCTTAACCATTGAAATACAAG
>Csa5G092920.2
TAGAACTACAGAGTGACACAATAAGCAGAGATTACAAGTTAGCTCAAGA
AACTCCCATGTTCAAAACATAACCACCATAGAAATTAACACCAAGAAGGA
AGAAAACAGTGTGCTTCAACTTCAAATAGCATCACTTCGATATTCCTACA
AATTAAAGAACTGCCCTGTCCCCGCAATTAAGCCCTAAAGAAGACAAAAC
TCATCAAATACACTTAAATTAAGTCTTTCCAAACCCAACAGTGAGGATTAT
AAATTAATTCCAACAAGATCTCGATATAACCAAAATGGGTTGATTTAACA
GCATACTGTTGATTGAAAAGTTCCAAAATTGGAAATTCCAGTATCCCACC
AACCAACTAAAAATTAGGGGTTCTATCTAATTAAAGCCCCTTTTCAATATA
CATATAGAAGGGTAAACATAAAGTAATCCGTTAACATAGATTTCCACCAT
AAAACCCTAAAACGGAGACAACTTTAAGATTCAAAATTCTCATCATACGG
TTACATAAACAAAAATCAGGGTTAGGAAATATATTGGAATAAACCGTCAG
AAACCAAAGTTGATCAGAAACAAAAAGGAATGAAAAAAATATAATAAAA
AAGTGGACATAGAAGAAGTATATATAAAGAACAAAGGATAGCAATATTT
ATATATGAAAATGTCAGTAATGGTCAAAGAGCACAAAGAAGGACCCCAT
ATATTGAGAGATGAGAGAGAGAGAGGAGACAAAGACAGAGAGAGAGAG
AGAGAGAAGGAGGAGGAGGAGAGAGAAAACTTACTTGGGTGGGTGTAAA
CGGCACTTTAGGGTTTTTGCCGGAGGAGAGAGAAACTGAAAGGGAGAGA
GAGAGAGAGAAAGGGAAGGAAAAGGAAAAGAAGGAGAAAAAGTGGGTG
AAAAAGGAAAGGGTTCTTTTGATATATATATATATAATATGTTATTTATCT
TATGAGTTTATGAGTACGAGAGAAATAGAGAAGGTTGTGACAAAAATCTT
CCAACCTAAATTAATTTATCATTATCTTTATGGGATCAATTCTTTTCTCTTT
TCTCTTTTTTCTTTTTTTGAAGATATCATTATTTGATTATTGTATTTTGGTGG

AAGTTTGATCAAAGAGAATGTTTTGGTTGCATTTACTCTCCCTTTTAAATA
TCTTTTTTTTCTTTTCTTTTCTTTTTTCTAATTCTTATTTTAATTTTCATTTGC
AAACTTAATTCAACTTAATATTAGTTTCTAGTGAGTTGTGCATCTCTCCATT
GCGCCAATTTTTAATTTTTAAAATATAGTATTGAATTTACTATCCAAACCA
ATCATTTTTATATTTTTATTTTTATCACTACATAAAATCCAACAATTCTTAA
CCTCAAATAAATGTCGAAAATTAATAGGTCGTGATATCCTACTATTTCAGA
CACTTGGAAAGTACTGTCAGTATACGGGTGCAATCCCAACGTCTTAATTAT
AACGTCGGAACTGATCCACTAATATATTTTTTTAAAATTTTATTCACGTTTT
GCCGACGTAATGAAAAAACGACCGTGAAAGTAAAAAGATTATTTAATTAT
TTAACTTCTTCTGACAACTAGGTAAGACTCGTCGAAGAAGCTAAAAATAA
TTATTTGATTATTCCACTTTTCCCAATATCTAGGCAAAATTCGTCACAAAA
AAATGGAAATAGTTAATTTAGTTGTTTAACTCTCTTGGATGTCTACGTTTA
CTGTGTCGGAGAAAATTAAATAGTTAATTAAATTGTTTAAATTTTCTTGAC
GTCATGCAGGAACGTCTTGAAAACTCCAACTAATATCGACGCCATTAGTG
AAATATCAACATGGGTTGAAACTATCTCCAATGTTTCACTTTATGACATTG
GAAATAACGTGCATTTTCAACATTTTTGAGATGCATTGAAAAATCCTTTGT
TCATTTTAAGTTATCTCCAATATCAAAGGTTTGTGGTACTTAGTAAGACAA
TAAGAGTGAAGGAACATGAAGACAATAGAAAT
>Csa7G007860.1
ATCCAAAGATTTTCTTAAAATTAAAATATAATATTTTTATTCTCATCAACTT
CTTTTTAAATTTTTTTTTAATAATTTAAGAAAAAGAAAGAAAGAAAACGA
GGGGCGAAAACAAACTTCAAAACGGCAAAGGAAAAACGCGTTTTAGGGG
GTTAGACTTTTAATCCAGTTTAAAGGAAGAAAGAAAAAAAAAAATATATA
TTTTTCCTTACAAACGACGTCGTAAGGAATTGGAAGCCAACGGGGTCGTT
TTCGGATGAGAATACACATCAAAACGAAGAAGAAGGAAGGAAGGAAGGA
AGGAAGGAAGTAGGCTTGCTACTCACCACCATTCTTTCTCTCTCAGTTCTC
TCTTCAATCTCCGACTCTATCAAATCTTCACCAAATCTCTTCCAAATTCTTT
TCAGGTCAGCATTCCTTCTTCTCCATTGCATCTGTTGTTGTAAGTTTTCCTT
TCCTCCATTCCAACTTCCTCTCTTTCTTCTCATCATCCTCCATTTTTCCCCCT
CTCCCTTTTCAAATTTCTTTCTCCTCCTCCTCCTCCTCCTCCTCCTAAATCCT
ATCCTTTTCTCACTCCTACCTCGCCTATATTCTTTTTCCTCCATTCTCTCTTT
GTTCTTCTTCCTTTCATGTTTTTTTCCCTCCTTTTAGGTTTTGTTCGAGGAGA
TTGGGCTGCTGCTCCTTTTTTTTTTTTTCGGTATGATTTATTCCTTTTTTTGAA
TCAACTCCTATTTTTAGCATTTTTTTGTGGTAAATTTTACTGTCTTTGTATG
TTTAATGCCAAGGAAATAGTGGAAAACATATGTGTGTGTGTGTGTGTGTG
TGTGTGAATGTGAGTGTCAATGTCATTTCTCTATATTTTGTAAAGTTTTTAA
CATTTTTTGTTTTTATTTGTTTAGCTTGTGTTATATGTTTTGTGTCTAGAGTT
TTGAGGAAATTGTTGTGGACTTAGTTTTGGTTGGACTTTTATTTATGAGTA
TTGTTTCTATGTTTGGTAAAAGATTGTTTATTTATGTTTTTGTAAGGTAAAG
ATGAGTAAATAATGGTTACGAGTCTGACTGTTGATTGTTTTTTTTATTTTAA
TTTATGCCGTTTTGTTTCGTCTTGTGTTCAGAGCTGTGAAGAAATTGGTAT
AGACTTGAATAAGTGAAGTTGATTATCCCTTGATGTTATAAGTAAGATGCT
GCTTCGGAATATGACCTTAAAATTTCTTCGGTGGATGGTAATGACCAGCTT
TCATTAAACTTAAGCTGAAATGGAAGAACCTACATACTCTTAACATGGTTT

TAAAGAGACTCAAATTGCCAGACCAGGCTTCTGTTTATGCTTTATCTTTGA
AAGAGCAATCCAATGAGAACCATTAAAGATGTATGAAAGTTTGATTGATT
AATGGCGTACTTGAATGTTTGTCTCCTATTTTAATTTGTGTCCATTATTTGG
ACAATCATAGTATTGTAGGCTCTTTTTTTCTTAGCTCTTGATAGTAAGTGTT
TGAATTGATCCCCCAAGATATGCTGGATGTGACCGTGCTGGTACCTCCTGT
TATCCAAACTTATGATCTTTGCTTTGTCAATGAAAAGAGACTGTATCATTT
TTAATTATTTGCTTGATGTAGCTGAATGAATTCATCGTAGTGTGGGGTGAT
TCCAATCTTCTTTATGATTTTACTGTAGTTCTAACATGTCTTTATATTGACC
AAAAGATAGTCTCCTTTCAGTTTAGGTTAATTAATGTAGGTTCAATTTTTC
TTTTAAACTTTTTGTGACTTTGAACTACCACTCATTCTTAGTTGTTGAGGTT
TTATTTCACTTTAGTTTCTATCTTTCCTGTTATGCCTTGTTGATGCTTCCCAT
TATCTAAATCTTGCTGATGTTTTCAATTTCATGTAAAATCAGAGTAGATGA
TTATGAGTTGGAAGGTTAGTTGATGGTGAAATTTTTTTGAATCAAACTCCA
ATCAGCATTCTGAATCCATTCGCCTTGGTAGTTATTTTTTGGGACT
>Csa2G365700.1
AAAAGAAAAAAAAAGAAAAAGAAAAAGAAAAAGGAAAGAGAGAGAGA
GAGAGAGAGAGAGATTCCTTTGTCCCTCTGCTCTGCTTACAACTGTTCTTT
CCACAGATTTTCAATCCTTTTCTTTTCTCCATCTTCATCTCTTTTCCTTTTTC
TTCTTCTTTCTTCAGTTCTTCATCTCTCCTTCTCACTGGTAAGCTTTCATTTC
TCTTTCCCTTTTGATTTCTTATATGTTTTTCCCTTTTCCCTAATCCCACTTTC
TATTTTGGTTTTTTATTTCCATTTCCTGCTTTTTACTCCTTTGAATCATATCT
GTTTCTATCACTTCTTCCTTATTCTATCTTTCTCCTGCTTACTCTCACTCCAA
TCTTTGTGAAATCTGCTTTAATTTACTGCTTATTTGCTGTTTTTGAAACTTG
CTGTGTGATTTGTGGCGTTTTTTTTTCTTCTAGGGTTTCGTTTGCTTCTGGTT
GTTATTGTTCTTTTTAGCTCAGAATTTGTTGTGGGGTTGTTTTATTTTTCGTT
TTGAGTTGAGGGTTCTGCTTTAAGCTCACGGCTCGAAGCTGAAAAGTTGA
CTCCCGTTTACTTTCCTGGTTATAGTAATTAAAGAATCGGATCTTTATGAG
TTTTGCTTTCTTCATAATCTTTTAGCCCTTTTTGCCCTATTTTTCAGCTGGTT
TGAGCTCGTTGAAACCGATGAATACGCTTTTTGTGACTGCTTTGACTTGAT
TGTTTTTGCTGTATCTACTGAATTTTTGTACTGGAAACTAAGGCAGGAGAG
GGAAATTACAGAAACATTGTTGGGGTTTATTTATTTTTGAAGTGTGATTAA
TTTATGTTGAATTTGATTGTTTAGATGATCTGATGGAGGATGAGATGGGGA
AGGTGCGTCAGCCATTTGAGCTGACTGGAGGTCGAAATTTCTAACGATGG
TTTCTTAAGTGGATTCTTCGTTTGGTCTTCTGATTCCAGGTGAAGTATCTAC
TGCTCCGAACTCACGTACATCTTGTTTCTGCTTATGATTACTTGCTTAGACT
GTGGATAAAGTGCTCTGCTGCTTAACTGTATTACTTCATACCTGAGCTTTA
ATTCCACAAGACAGTTATTTCTCTCTCTCGATTGTTTATTGTTGCCCAGAA
GTTGTAGAGGTGTTTTTAGATCTTCCCTTTTATGGATGTGTATATGTATACA
ATTTTTGCAACCCTAGTTGGTCAGGTCGGTTAGTGTCTTCTTTGTTTGGTCA
GAATTTTGAATGTTTCTCTTAATGGAAGTCAATAGACTTAACTTGTGGTAG
TAACTTGTGTATACAATAAAGGGAGTGATGCGTCCGTTGTTGGAATGATG
TTGTATTGATTGCCACCTCCTCGATCAATTAGAGTTGGTTCTTTAAGGGGT
TATATTTGGCTTTGGCTTGTAGTTTTATCTGAATTTTTTATTTGTGCCACCG
GAGTTTGATTTTGAATTTGAATAAACTAATATTATTCTCTTTATTTAGAGA

GTGAACGGTATCAAACATCAATGTCAAGAAAAGTGAAATTACATCGTCAA
TTTATTTGAAAAATAAAGTTATAAACACACTTTCACTCCTAAGTTTCTTTA
ACTTTGACTTTGTTCTTAGAATTTAAAAATGAGAGAAAGTAAATATAAATT
TCAAAGATAAACTAAAATAAAACTATTATAAGTTGGAGCTCCGTTTGGGA
TGATTGTTCTCACATTTTTCTTTTACTCATGGTTTTGATTGTTGGCTCATTTT
TAATAACAAAAATATATATTTTTGATTTTCTCTTTTTGCGCTGAATAGAAG
TATTTCTTTTGAGGTTCACCAATATGTGAATATTTTATTTGAGACAGCATTT
TCTTAGCTTGCATTCTTTCTCTTCATAGTGTATATCATAGTTAGGTAACATA
TTGGTAACATTTAAGCATATGAATGATTGCCATTTTGTTTGCAGTGGAGCT
TTTTCATTACTGTATCTGTGTTGGTTATTCGTTTTGGAGTCACAA
