# Supplementary material for: Genome-Wide Identification and Characterization of Cucumber BPC Transcription Factors and Their Responses to Abiotic Stresses and Exogenous Phytohormones
Source: Int J Mol Sci. 2019 Oct 11;20(20):5048. doi: 10.3390/ijms20205048 (PMC6829308; doi:10.3390/ijms20205048)
Supplement: Supplementary file 1 [file ijms-20-05048-s001.zip › Additional file 3.pdf]

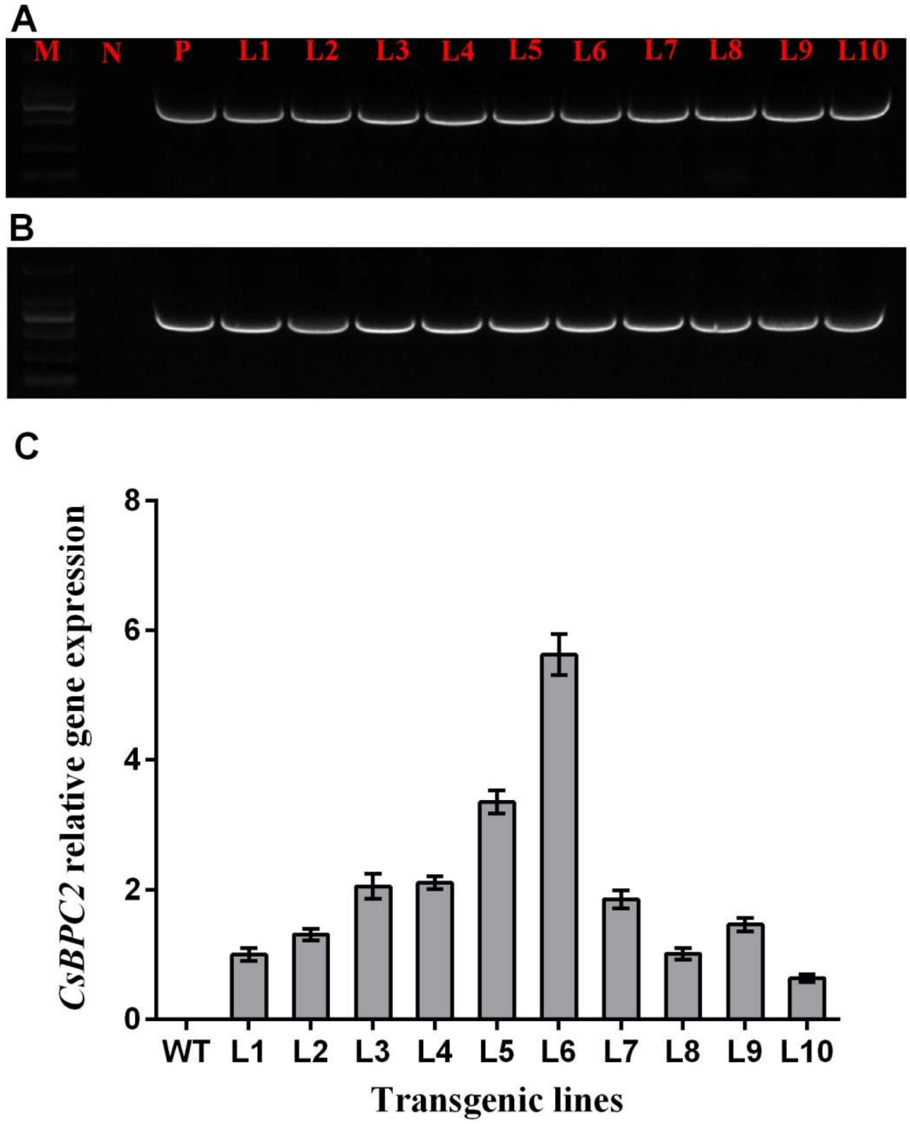

Identification of transgenic tobacco plants by PCR and qRT-PCR.

(A) and (B) PCR identification of transgenic tobacco plants. Primers for *Hyg* (B) and *CsBPC2* CDS amplification (A) were used to identify the transgenic lines. L1, L2, L3 etc represent ten independent T0 transgenic lines; M: DL 2000™ DNA marker; N: non- transgenic plants (negative control); P: plasmid DNA (positive control). (C) qRT-PCR analysis of *CsBPC2* transcript level in T0 transgenic lines. *CsBPC2* specific primers was used to detect the transcript level, and tobacco NtActin primers was used as a control. The experiment was repeated in triplicate per sample.
